# Positive Associations Between Nutrition Policy and Food Environment Scores on Military Installations

**DOI:** 10.3390/nu18121976

**Published:** 2026-06-18

**Authors:** Sarah J. Hinman, Emma N. Alitz, Katie M. Kirkpatrick, Jessica L. Kegel, Melissa A. Rittenhouse, Jonathan M. Scott

**Affiliations:** 1 Center for Advanced Research for Military Optimization, Readiness, and Rehabilitation, Uniformed Services University, 4301 Jones Bridge Road, Bethesda, MD 20814, USA; emma.alitz.ctr@usuhs.edu (E.N.A.); katie.kirkpatrick.ctr@usuhs.edu (K.M.K.); jonathan.scott@usuhs.edu (J.M.S.); 2 Henry M. Jackson Foundation for the Advancement of Military Medicine, Inc., 6720A Rockledge Drive, Bethesda, MD 20817, USA

**Keywords:** food environment, nutrition policy, military nutrition environment, military

## Abstract

Background/Objectives: Due to the rise in chronic disease in the United States, several policies, guidelines, and programs have focused on promoting healthy eating. This includes those aimed at service members, who may struggle to find nutritious food options on military bases. The Military Nutrition Environment Assessment Tool (mNEAT) assesses the access, availability, and promotion of nutritious foods and beverages on military bases. Military services vary in their policies related to the food environment, including the use of mNEAT, on their installations. The aim of this article is to examine the difference in mNEAT scores for those military services with a comprehensive mNEAT policy compared to those with a partial policy. Methods: Installations with mNEAT scores from October 2021 to September 2024 were separated into two groups: full-policy group (Air Force, Space Force, n_f_ = 76) and partial-policy group (Army, Navy, Marine Corps, n_p_ = 69). Food venue types assessed include: commissary (n_f_ = 67, n_p_ = 44); fast-food (n_f_ = 63, n_p_ = 48); vending (n_f_ = 42, n_p_ = 18); express stores (n_f_ = 65, n_p_ = 50); food truck (n_f_ = 42, n_p_ = 24); dining facility/galley (n_f_ = 51, n_p_ = 40); Morale, Welfare, and Recreation (MWR) food service venues (n_f_ = 56, n_p_ = 38). Installation-level venue types assessed include community (n_f_ = 56, n_p_ = 24) and worksite (n_f_ = 49, n_p_ = 20). Installations were separated into venue-type variety groups according to how many different venue types they assessed: low (1–4 venue types, n = 47), moderate (5–7 venue types, n = 48), and high (8–9 venue types, n = 50). Results: The full-policy group had higher total installation mNEAT scores on average than the partial-policy group (54.6% vs 50.4%, *p* = 0.034), but results were no longer significant when venue type assessment variety was added as a covariate (*p* = 0.074). In addition, the full-policy group had higher scores (*p* ≤ 0.05) for the following venue types: dining facility/galley, MWR, fast-food, express, and community. In the moderate (*p* = 0.012) and high (*p* = 0.022) venue-type variety groups, the full-policy group had significantly higher mNEAT scores, but not for the low venue-type variety group (*p* = 0.547). Conclusions: Higher mNEAT scores for full-policy installations may indicate a more supportive food environment than those in the partial-policy group, when ≥5 food venue types were assessed. Military services without a comprehensive mNEAT policy can consider modifying or implementing one to support easier access and availability of nutritious options. This public health strategy can support service members’ health and performance and can be applied to similar settings, such as universities and worksites.

## 1. Introduction

Given the impact of nutrition on chronic disease risk [1], there is interest in guiding Americans to identify and choose nutritious foods that contribute to health and well-being [2,3,4]. Over time, nutrition guidelines and mandates, public health programs, and policies have been established to improve the eating habits and, therefore, the health of Americans. Environment-level nutrition policies—which include nutrition standards, pricing, promotion, and labeling in schools, workplaces, and communities—can improve diet quality as well as physical and mental health outcomes [5,6,7,8,9].

While there are established links between the impact of nutrition policies and policy-driven interventions and improvement of diet and health outcomes among the general population [7,8,9], there is less knowledge about these outcomes in military settings. The United States military is facing a growing challenge to both recruit and retain service members, with obesity being the leading cause of recruitment disqualifications [10]. In addition, obesity contributes to early attrition, the inability to meet physical fitness standards, and a $1.35 billion cost burden for the Department of War (DoW) annually [10]. Improving the nutrition habits of service members is critical to their health, performance, and military readiness [11,12,13]. Thus, policies to assess and improve the food environment are a critical public health strategy to support service members.

The current food environment on U.S. military installations may not optimally support service members [14,15,16,17]. The military food environment includes everywhere service members can access food and beverages on a military installation or ship, including dining facilities (DFACs)/galleys, mess halls, fast-food restaurants, vending machines, convenience stores, and food trucks. DFACs, galleys, and mess halls use appropriated funds to buy and procure food. These facilities are required to follow DoW policies and menu standards to provide a certain amount of healthy foods and beverages at each meal [18,19]. Other types of food venues, such as fast-food restaurants and convenience stores, do not use appropriated funds and therefore are not required to follow the same DoW policies.

Service members have expressed challenges with finding healthy food options on military installations [15,20]. Army Soldiers have identified the proximity of numerous fast-food venues, which often lack nutritious options, and the higher cost of nutritious options prohibiting them from choosing them on their installation [15]. Similarly, Navy Sailors have reported that healthy food was difficult to find on their installation, and they often purchased food from convenience stores, vending machines, coffee shops, and fast-food establishments [16].

DoW nutrition and health-promotion experts created the Military Nutrition Environment Assessment Tool (mNEAT) for local health and food professionals to assess the food environment on military installations and ships. This web-based app provides a snapshot of the availability, access, and promotion of nutritious foods and beverages at nine different types of food venues. The DFAC/galley assessment also evaluates adherence to key components of DoW policies, including menu standards and nutrition program requirements. The mNEAT Facilitator’s Guide [21], which acts as a how-to handbook for users of all miliary services, encourages users to leverage their mNEAT results to identify areas of success and opportunities for improvement at their local installation. It recommends users assess at least one venue of each venue type, two for fast-food if there are multiple different chains located on the installation. The Facilitator’s Guide then encourages users to create a plan to improve their food environment that targets a lower-scoring area in collaboration with local installation or ship leadership and other stakeholders. Another recommended best practice is for users to reassess local installations annually and note any changes in scores.

Although mNEAT is a military-focused tool, the requirements for its use vary by military service. The Department of the Air Force, which includes the Air Force and Space Force, has a comprehensive policy that specifically references mNEAT and requires the Health Promotion Working Group on each installation to complete an mNEAT assessment annually [22]. The local Health Promotion Working Group is instructed to brief installation leadership on mNEAT scores and develop and implement a plan to improve the food environment. A checklist completed by unit leadership determines compliance with this policy by confirming the Health Promotion Working Group completes all the required elements annually. Additionally, all food facilities are directed to “work to ensure” all dining options on the installation include healthy items and meet the minimum criteria for mNEAT assessment [22]. As such, assessing each venue is necessary to verify whether these criteria are consistently met across all facilities.

In contrast, the Army, Navy, and Marine Corps policies are not as comprehensive as the Air Force policy. For example, Army policy states that mNEAT be used to measure accessibility of healthy foods and to prioritize changes to improve healthy eating, but it does not provide guidance on when mNEAT assessment should be conducted or by whom [23]. Navy and Marine Corps policy simply states that the food environment be assessed, but not how or what to do with the results [24]. Neither policy includes a method of checking compliance.

The aim of this article is to compare mNEAT scores between military services that have a comprehensive policy (that requires mNEAT assessment, an improvement plan, a quality control check, requirements for minimum mNEAT scoring, and the involvement of installation leadership) to those military services with a less specific policy related to food environment assessment.

## 2. Materials and Methods

### 2.1. mNEAT Assessment Scoring

mNEAT can be used to assess nine venue types: commissaries; fast-food establishments; vending; express stores; food trucks; worksites; DFACs/galleys; Morale, Welfare, and Recreation (MWR) food service venues; and the installation community (Table 1). mNEAT generates venue scores for food policy, food availability, and behavioral design for commissaries, fast-food, express stores, food trucks, DFACs/galleys, and MWR venues. Food policy addresses which policies are enacted at an individual venue, including policies established at the venue, installation, or DoW level. Food availability includes the availability of specific healthy items (e.g., whole-grain options, fresh fruit). Finally, behavioral design is focused on promoting nutritious food (e.g., posters, promotional materials). Commissary venues have an additional score for community involvement. The community, worksite, and vending venue types are scored differently than the other venue types. Community questions are focused on installation-based initiatives and policies rather than food venues. Worksite questions assess policies at worksites (e.g., the Military Treatment Facility/hospital). The vending assessment asks about vending contracts on the installation, if they require healthier options, and whether they promote healthy options. See Appendix A for a sample of mNEAT assessments.

The scores for all venues of the same type are added together and divided by the maximum possible score to create a percentage score for each venue type. These venue type scores are then combined to create a percentage score for the overall installation. The higher the scores, the more supportive the food environment of the installation. It is up to the assessor at each installation to decide what to assess, which could be anywhere from a single venue to all venues on their installation, depending on time and available resources.

### 2.2. Study Sample

The mNEAT web-based app and database contains data from each DoW service (Air Force, Army, Marine Corps, Navy, and Space Force). A total of 145 installations were included in the analysis based on completion of at least one venue assessment between 1 October 2021, and 30 September 2024. See Table 2 for distribution by service. The installations were placed into one of two policy-group categories (Table 2):

- Full-policy group (n_f_): The installation’s military service has a comprehensive policy requiring and describing use of mNEAT.
- Partial-policy group (n_p_): The installation’s military service has a partial assessment policy that does not provide details of assessment.

Air Force and Space Force installations are in the full-policy group (52.4%; n = 76), and Army, Navy, and Marine Corps installations make up the partial-policy group (47.6%, n = 69).

While it is not directly specified in any official mNEAT policy, it is recommended by the Facilitator’s Guide to assess one location for each of the food venue types for a more comprehensive evaluation of the food environment. Only 20 of 145 (13.8%) installations assessed at least one location from each venue type. Nevertheless, most installations demonstrated broad assessment coverage: more than two-thirds assessed at least five venue types, a proportion that increased from just over 50% among partial-policy installations to nearly 83% among full-policy installations.

### 2.3. Statistical Analysis

Data preparation and analyses were completed using IBM SPSS Statistics for Windows, version 30.0.0.0 (Armonk, NY, USA: IBM Corp). Frequencies were used to summarize the administrative characteristics of all military installations in the study sample. Measures of central tendency (i.e., mean and standard deviation for normally distributed data; median and interquartile range [IQR] for non-normally distributed data) were computed for mNEAT total installation scores and venue type scores. All statistics were computed using the last assessment completed at each installation between 1 October 2021 and 30 September 2024.

Independent samples *t*-tests were conducted to assess differences between the two policy groups in average mNEAT total installation scores and venue type scores. In instances where the assumption of normality was violated (determined by evaluating skewness, kurtosis, and the Shapiro–Wilk test), non-parametric Mann–Whitney U tests were used instead of *t*-tests. To determine effect size, Cohen’s d was calculated for parametric analyses, and Glass rank biserial correlation coefficient (r_rb_) was calculated for non-parametric analyses [25].

Hierarchical linear regressions were computed to assess if comprehensive policies that require mNEAT at an installation significantly predicted mNEAT total installation scores while controlling for recommended venue-type variety among the installation assessments (at least one venue assessed for each of the nine venue types). To further explore the effect of venue-type variety on mNEAT scores, installations were categorized into three groups: low venue-type variety (1–4 venue types assessed, n = 47), moderate venue-type variety (5–7 venue types assessed, n = 48), and high venue-type variety (8–9 venues assessed, n = 50). Linear regressions were run for each of the three groups to understand the effect of policy on total installation mNEAT scores at each level of venue-type variety. For all regression models, standardized and unstandardized, 95% confidence intervals and beta coefficients were computed. The partial-policy group was used as the reference group in the analysis for these models.

## 3. Results

Regarding the overall scores for installations (Table 3), the full-policy group had higher total installation mNEAT scores on average than the partial-policy group (54.6% vs. 50.4%). The full-policy group also had higher mNEAT scores on the DFAC (Table 3, 61.8% vs. 54.3%), MWR (Table 3, 37.6% vs. 30.5%), fast-food (Table 4, 40.1% vs 33.0%), express (Table 4, 70% vs 58.7%), and community (Table 4, 83.3% vs 56.4%) assessments.

Hierarchical linear regression results indicate that mNEAT policy is a significant predictor of mNEAT total installation scores, with the full-policy group obtaining overall higher scores than the partial-policy group (B (95% CI) = 4.15 (0.32–7.99); SE(B) = 1.94; *p* = 0.034; R^2^_adj_ = 0.02). However, when venue-type variety (the number of different venue types assessed) was added as a covariate, the difference was no longer significant (B (95% CI) = 3.53 (–0.58–7.63); SE(B) = 2.08; *p* = 0.074; R^2^_adj_ = 0.02).

When the installations were split into venue-type variety groups, the linear regression results signify higher mNEAT total installation scores among the full-policy group for the moderate (B (95% CI) = 7.72 (1.75–13.69); SE(B) = 2.08; *p* = 0.012; R^2^_adj_ = 0.11) and high (B (95% CI) = 5.54 (0.82–10.26); SE(B) = 2.08; *p* = 0.022; R^2^_adj_ = 0.09) venue-type variety groups but not the low group (B (95% CI) = −3.1 (−13.38–7.18); SE(B) = 5.10; *p* = 0.547; R^2^_adj_ = 0.09).

## 4. Discussion

mNEAT scores were higher for the full-policy installations compared to partial-policy installations. This suggests at least an association between full mNEAT policies and food environments that may be more supportive of healthy food access, availability, and promotion. A comprehensive, full mNEAT policy requires use of mNEAT and describes how to use it, including involving installation leadership, implementing an improvement plan, and passing an annual check [22]. In addition, it dictates that food venues must work to ensure all new dining options on installations meet the minimum criteria for their respective mNEAT assessment type [22], which suggests an mNEAT assessment is required at each location to determine scoring. Of the comprehensive policy requirements, leadership involvement, in particular, is a driver for change in the food environment on military installations [26]. Installation leadership can support the food environment at the installation level through policies related to healthy-eating initiatives, endorsing improvements to the food environment, and/or supporting nutrition education [27]. Additionally, the requirement for annual improvement plans also targets a method to increase the scores each year, which could lead to higher scores for the full-policy group [28]. Taken together, the findings suggest that a comprehensive policy plays a role in creating a more supportive food environment than partial policies where fewer details regarding how to assess and improve the food environment are provided.

However, a positive association between policy and the mNEAT scores is absent for installations with low venue-type variety levels. At these installations, a lack of variety in venue type assessments might not provide a comprehensive installation score. The presence of an association between policy and mNEAT scores in the moderate and high venue-type variety groups indicates that this association may hold for most installations, that the low venue-type variety group may lack the quantity and variety of assessments required to see it. Future studies that target using mNEAT scores to assess the food environment should look at requiring installations to have a minimum number of different venue type assessments or ensure equal venue type contributions to the total installation score.

Current findings indicate emerging evidence of a policy effect, particularly within the moderate and high venue-type variety groups, where associations were stronger. While the overall variance explained was modest, the pattern suggests that higher venue-type variety may enhance the detectability and consistency of policy effects across venues. This indicates that the relationship between policy and total installation scores is sensitive to other factors that warrant further investigation. Since assessors independently determine which venues to evaluate, installations may not be represented by a comparable set of venue types when few different venue types are assessed. If lower-scoring venue types are less likely to be assessed, total installation scores may be artificially inflated regardless of policy status. Consequently, differences in venue selection represent a potential source of selection bias and may affect the comparability of installation-level scores, posing a threat to the internal validity of the analysis. However, this concern may be less pronounced among installations with a greater variety of venue types assessed. Broader venue type coverage is more likely to capture the diversity of the installation food environment and reduce the influence of selective assessment of individual venue types. Consistent with this interpretation, statistically significant differences between policy groups were observed in the moderate and high venue-type variety strata, suggesting that analyses based on a larger and more diverse set of assessed venues may provide more representative estimates of policy-related differences. Alternatively, smaller installations may have limited options for certain venue types, impacting their ability to have their scores compared equally to large installations with many venues of a certain type. Some installations are located in rural areas with little food options off-installation and others in urban environments with many off-base options, which may affect the amount and types of food venues available on the installation. Additionally, rural installations may struggle to supply adequate food due to longer shipping distance, availability from local vendors and/or lack of infrastructure. The relationship between policy, venue-type assessment variety, installation environmental characteristics, and total installation scores need to be investigated further.

The full-policy group scored higher on the DFAC, community, MWR, fast-food, and express venue types than the partial-policy group. Although DFACs and galleys fall under DoW menu standards and nutrition program policies [18,19], additional comprehensive policies that require annual mNEAT assessment and improvement may make it more likely that those locations comply with the standards and requirements, since the mNEAT assesses some of their components (see Appendix A.1). Also, DFACs are often chosen for improvement plans because they are usually the easiest places to intervene, due to the control that installation leadership has over their operation [17], their governing standards and requirements [18,19], and a pre-established relationship the assessor may have with DFAC management. The questions in the community venue mNEAT assessment directly evaluate some of the requirements of the comprehensive mNEAT policy (e.g., assessing the installation’s food environment and developing an improvement plan; see Appendix A.2), which might explain the full-policy group’s higher scores for their community venues. MWR food venues are often grills, bowling alleys, golf course restaurants, or other establishments that specialize in “American style” food that tends to be of less nutritional value. Due to this, these venues have lower mNEAT scores and likely seem like a good place to target improvement plans. These venues are supported by a combination of self-sustaining revenue generation and taxpayer money [29] and may be more amenable to making changes in their menu or offerings as they are not solely reliant on their profits. Similarly to MWR venues, fast-food venues may provide a good target for intervention due to the low mNEAT scores. Installations may have some control over the types of fast-food venues located there [30], which may incentivize full-policy installations to prefer higher-scoring venues since the comprehensive mNEAT policy states food venues work to meet mNEAT scoring standards. Express venues are typically similar to convenience stores, with a mixture of fresh food, frozen food, and packaged items. Air Force, Space Force, and Army express are run by the same organization (Army and Air Force Exchange Service), and these venues have a program for stocking and promotion of healthy items [31]. The Navy and Marine Corps have different companies that run their express locations (Navy Exchange Service Command and Marine Corps Community Services respectively). The division of full- and partial-policy services across organizations may account for variations in scoring, given that these distinct entities may have different food-related policies. Further research is needed to understand the effect of other food and wellness policies at different levels on mNEAT scores.

Commissary, worksite, food truck, and vending venue type assessment scored similarly between the full-policy and partial-policy groups. The Defense Commissary Agency (DeCA) institutes nutrition policies that commissaries across all military services must follow, such as identifying and labeling healthy items with a Dietitian Approved Thumb [32], which may explain why the commissary mNEAT scores were similar between policy groups. The mean for worksite mNEAT scores in both groups was zero, as most installations had no installation-level policy related to healthy food and beverage offerings at meetings or at worksites (see Appendix A.3). This may be due to the difficulties (real and perceived) associated with creating and implementing a worksite healthy food policy seen even outside the military [33]. Additionally, senior military leaders rotate every couple of years making it difficult to develop and implement a worksite policy in a limited time. Food trucks and vending were among the least assessed venue types, as more data is gained, differences in the scores between groups may be shown. The similarity in food truck and vending scores may also be because full-policy group installations do not often target them for score improvement. Food trucks are often run by individuals or private companies without any healthy food policies or who may believe that healthy options are not profitable or desirable by customers. Vending on military bases is contract based and if the contract does not state that healthy items must be supplied, the contracted company or individual may decide not to add such items [30]. Understanding the effect of improvement plans and food and beverage contracts on mNEAT scores is needed to elucidate this further.

mNEAT focuses on the assessment of the military food environment and gives a score that estimates how health-supporting it is for the service members that live and work there. It does not directly assess how that food environment improves the health of service members. Studies on the food environment’s impact on individual health are mixed in civilian studies [34,35], and might be more closely related to the venue types available [36]. However, environmental-level policy can be an effective way to improve health outcomes in individuals [7,8,9]. Taken together, this may imply that policy is a part of the impact the food environment can have on health, and that there needs to be support from leadership or government to drive health outcomes. Certain venue types, like grocery stores and fast-food, might be a more effective way to influence individual health, positively or negatively [36]. These venue types on military installations (commissaries and fast-food respectively) may also have an increased impact on service member health and total installation mNEAT scores. Furthermore, changes at the venue level for these establishments may provide a more meaningful interpretation than differences in overall installation scores, given that mNEAT is structured to assess and compare similar types of venues across installations. Future research should examine how installation-level mNEAT scores relate to service member purchasing behaviors, dietary patterns, and health outcomes to better establish the practical significance of observed score differences and to identify which components of the military food environment have the greatest impact.

This analysis has some limitations. The assessors may or may not have been trained on how to complete an mNEAT assessment, and each assessor decides which venues to assess and how many of each venue type. While the facilitators guide recommends at least one of each venue type, if that is not part of policy it is unlikely to happen. As a result, low-scoring venues may have been skipped, or other venue types may be over- or under-represented in the sample, introducing selection bias to the total installation mNEAT scores. Assessors are encouraged to assess at least one venue of each venue type, but assessment of all venues at an installation is needed to fully understand its food environment. In addition, the version of mNEAT that was in use at the time did not collect information on improvement plans during the analysis period. A recent update of the tool now allows users to design an improvement plan in the app and update the progress and outcomes of its implementation in their food environment. This information will be useful to track how those plans influence the scores at the venues where they are enacted.

Future studies should examine how improvement plans and other nutrition-related policies and initiatives influence the food environment on military installations, since mNEAT policy is only a part of the overall picture. Focusing on certain venue types and looking closer at what drives higher mNEAT scores may be impactful in creating new policies or guidelines for similar venues on other military installations. Investigation into how and why certain venues are targeted for inclusion in mNEAT assessments and others are not may help understand the impact of venue-type assessment variety on mNEAT scores. On 29 December 2025, the department of the Navy updated their policy to name the mNEAT as a recommended tool to measure the food environment [37]. The new policy was posted after the data collection and analysis detailed in this manuscript. This provides an opportunity to examine the effect this new level of policy has on Navy and Marine Corps food environments. Future research can expand on these initiatives to further evaluate the practicality of setting a benchmark for the number of venues required to significantly influence installation mNEAT scores.

The overarching purpose of assessing and implementing food environment policies on military installations is to support service member health, performance, and military readiness. While tools such as mNEAT evaluate the extent to which the food environment aligns with evidence-based nutrition standards, these assessments are intended to inform policies and practices that create conditions conducive to healthier choices and, ultimately, a more mission-ready force.

## 5. Conclusions

The presence of a comprehensive policy requiring an annual review of a military installation’s food environment using mNEAT assessment suggests an increase in the availability and accessibility of nutritious foods and beverages on military installations compared to those military services with partial policy, provided enough venue types are assessed. The policy requirements of an improvement plan, a quality control check in the form of an annual review, requirements for minimum mNEAT scoring, and involvement of installation leadership are likely what makes it effective. Military services that do not have a comprehensive mNEAT policy could consider implementing it as a requirement to build a more supportive food environment to help service members maintain and improve their health and performance. While this study focused on the military food environment, it demonstrates the effectiveness of a comprehensive policy that requires and describes the use of a tool, such as mNEAT, to assess and improve the comparable policy-led food environments of universities, schools, care facilities, and similar establishments.

## Figures and Tables

**Table 1:** Food service venue types assessed by mNEAT (Military Nutrition Environment Assessment Tool).

Venue Type	Description
Commissary	Grocery store
Community	Installation-level questions about policies, partnerships, and community nutrition initiatives (e.g., community gardens, farmer’s markets)
Dining Facility (DFAC)/Galley	Cafeteria-style facility that provides meals to authorized personnel; operated with appropriated funds
Express	Small retail stores, such as convenience stores; military-run or contracted
Fast-food	Commercial fast-food restaurants
Food truck	Mobile food operations; commercial or military-run
Morale, Welfare, and Recreation (MWR)	Clubs, restaurants, bowling alleys, etc.; run by military-funded MWR or contracted
Vending	Vending contracts, including healthy-option policies
Worksite	Installation-level policy or guidelines for offering healthy food and beverages at meetings, worksites, snack bars, etc.

**Table 2:** Administrative characteristics of mNEAT assessment sites by policy group.

	Full-Policy (n_f_ = 76)		Partial-Policy (n_p_ = 69)		All Installations (n = 145)	
	n	%	n	%	n	%
**Military Service**						
Air Force	71	93.4%	—	—	71	49%
Space Force	5	6.6%	—	—	5	3.4%
Army	—	—	51	73.9%	51	35.2%
Navy	—	—	11	15.9%	11	7.6%
Marine Corps	—	—	7	10.1%	7	4.8%
**Venue Type Assessments**						
Commissary	67	—	44	—	111	—
Community	56	—	24	—	80	—
DFAC	51	—	40	—	91	—
Express	65	—	50	—	115	—
Fast-Food	63	—	48	—	111	—
Food Truck	42	—	24	—	66	—
MWR	56	—	38	—	94	—
Vending	42	—	18	—	60	—
Worksite	49	—	20	—	69	—
**Number of Venue Types Assessed**						
1	4	5.3%	12	17.4%	16	11%
2	1	1.3%	7	10.1%	8	5.5%
3	6	7.9%	8	11.6%	14	9.7%
4	2	2.6%	7	10.1%	9	6.2%
5	4	5.3%	6	8.7%	10	6.9%
6	12	15.8%	9	13%	21	14.5%
7	13	17.1%	4	5.8%	17	11.7%
8	21	27.6%	9	13%	30	20.7%
9	13	17.1%	7	10.1%	20	13.8%

n_f_ = number of installations in full-policy group. n_p_ = number of installations in partial-policy group. — = Not applicable.

**Table 3:** Independent samples *t*-tests for differences in mNEAT scores between full- and partial-policy groups.

Assessment Scores	Average Number of Venues Assessed (SD)	mNEAT Scores Mean (SD)		*t* (df)	*p*-Value	*d*
		Full-Policy Group (n_f_ = 76)	Partial-Policy Group (n_p_ = 69)			
Total Installation	12.2 (9.0)	54.6 (11.3)	50.4 (12.0)	−2.14	0.034 *	−0.36
Commissary	1.2 (0.5)	77 (15.1)	72.6 (12.7)	−1.61	0.11	−0.31
DFAC	2.1 (1.8)	61.8 (15.0)	54.3 (14.3)	−2.78	0.006 **	−0.51
MWR	2.5 (2.2)	37.6 (13.5)	30.5 (11.5)	−2.64	0.01 *	−0.55

Note: For parametric independent samples *t*-tests, mean (with standard deviation [SD]), *t*(df) (test statistic), and Cohen’s *d* (effect size) are reported. ** Statistical significance at *p* < 0.01. * Statistical significance at *p* < 0.05.

**Table 4:** Mann–Whitney U tests for differences in mNEAT scores between full- and partial-policy groups.

Assessment Scores	Average Number of Venues Assessed (SD)	mNEAT Score Median (IQR)		r_rb_
		Full-Policy Group (n_f_ = 76)	Partial-Policy Group (n_p_ = 69)	
Fast-Food	4.9 (4.2)	40.1 (15.9)	33.0 (16.9)	−0.29 **
Express	2.2 (1.9)	70 (20)	58.7 (18.9)	−0.34 **
Food Truck	2.3 (2.0)	22.4 (18.6)	21.6 (14.8)	0.06
Vending	1.1 (0.4)	48.8 (28.2)	36.6 (41.6)	−0.21
Community	1.1 (0.2)	83.3 (32.1)	56.4 (60.7)	−0.43 **
Worksite	1.1 (0.2)	0 (33.3)	0 (45.8)	0.05

Note: For non-parametric Mann–Whitney U tests, median (with interquartile range [IQR]), and Glass’s r_rb_ (effect size) are reported. ** Statistical significance at *p* < 0.01.

## Data Availability

The data presented in this study are available on request from the corresponding author due to restrictions associated with data collection on military installations.

## Abbreviations

The following abbreviations are used in this manuscript:

DOW	Department of War
DFAC	Dining Facility
mNEAT	Military Nutrition Environment Assessment Tool
MWR	Morale, Welfare, and Recreation
NY	New York
USA	United States of America
Corp	Corporation
IQR	Interquartile Range
SD	Standard Deviation
DF	Degrees of Freedom
CI	Confidence Interval
DeCA	Defense Commissary Agency

## Appendix A

The following are the mNEAT questions for assessment types: DFAC, community, worksite, and fast-food. The express, MWR, and vending assessment forms have been published elsewhere [28]. The assessments provide a sample of the types of questions on assessments across venue types; not all venue types were included for brevity’s sake.

### Appendix A.1

DFAC Assessment Form (This form was current as of the data collection, it was updated March 2025)
FOOD POLICY
1. What nutrition program is currently being followed at this location?
◯ G4G 1.0  ◯ G4G 1.5  ◯ G4G 2.0  ◯ G4G Army  ◯ Fueled to Fight  ◯ THOR3
◯ Other  ◯ NA  ◯ SFI-IMT
2. Are current training certificates for the nutrition education program (identified in question 1) available for the DFAC/Galley manager and subordinates employees for the current year?
◯ Not available  ◯ Available for Managers (but not the current year)
◯ Available for Managers (current year)
◯ Available for Managers and subordinates (but not the current year)
◯ Available for Managers and subordinates (current year)
3. Is there an annual training plan for nutrition education program to capture a rotational training, including new personnel?
◯ No annual plan   ◯ Annual Training Plan is in place, not followed
◯ Annual Training is in place, addresses rotational training incl training for new employees
4. Does this facility offer one or less deep fat fried items per day (main line and short order). This includes French Fries, main entrees, vegetables and starches
◯ Yes     ◯ No
FOOD AVAILABILITY
5. How many different healthy beverage options are available?
◯ 0     ◯ 1–5    ◯ 6–10   ◯ 11–15  ◯ ≥16
Breakfast
6.How many dry cereals with ≥2.5 g fiber and ≤10 g sugar/serving are available?
◯ 0     ◯ 1     ◯ 2     ◯ ≥3
7. How many hot cereals without added fat or sugar (or soup) are available?
◯ 0     ◯ 1     ◯ 2     ◯ ≥3
8. How many options of whole grain bread products (≥2 g fiber/serving) are available?
◯ 0     ◯ 1     ◯ 2     ◯ ≥3
9. How many healthier á la carte options (meat or vegetarian) are available on the hot/main line?
◯ 0     ◯ 1–2    ◯ 3–4    ◯ ≥5
10. How many healthier options (meat or vegetarian) are available at the grill/short order?
◯ 0     ◯ 1     ◯ 2     ◯ ≥3
11. How many healthier options are available at the breakfast/fitness bar?
◯ 0     ◯ 1–2    ◯ 3–4    ◯ ≥5
12. How many fresh, whole or precut fruits are available?
◯ 0     ◯ 1–2    ◯ 3–4    ◯ ≥5
Lunch or Dinner
13. How many healthier entrée options (meat or vegetarian) are available on the hot/main line?
◯ 0     ◯ 1     ◯ 2     ◯ 3     ◯ ≥4
14. How many healthier vegetable sides are available on the hot/main line?
◯ 0     ◯ 1     ◯ 2     ◯ ≥3   ◯ NA
15. How many healthier non-vegetables sides are available on the hot/main line?
◯ 0     ◯ 1     ◯ 2     ◯ ≥3
16. How many healthier options (meat or vegetarian) are available at the grill/short order?
◯ 0     ◯ 1     ◯ 2     ◯ ≥3
17. How many lean, low-fat deli meat or cuts from whole muscle meat options are available at the deli station for making sandwiches and wraps?
◯ 0     ◯ 1–2    ◯ 3–4    ◯ ≥5
18. How many whole grain bread/wrap options are available?
◯ 0     ◯ 1     ◯ 2     ◯ ≥3
19. How many options of healthier yogurts (<3 g fat, <30 g sugar, >5 g protein) are available?
◯ 0     ◯ 1     ◯ 2     ◯ ≥3
20. How many varieties of fresh, whole, or pre-cut fruits are available?
◯ 0     ◯ 1–2    ◯ 3–4    ◯ ≥5
Salad Options
21. For salad options, how many varieties of leafy greens are available?
◯ 0     ◯ 1     ◯ 2     ◯ ≥3
22. For salad options, how many plant-based toppings are available?
◯ 1–9    ◯ 10–11   ◯ 12–13   ◯ ≥14
23. For salad options, how many reduced fat dairy toppings are available?
◯ 0     ◯ 1     ◯ 2     ◯ ≥3
24. For salad options, how many lean protein toppings (with no added fat) are available?
◯ 0     ◯ 1–2    ◯ 3–4    ◯ ≥5
25. Are fresh toppings include at a minimum one item from each color category: (1) red, blue or purple produce, (2) orange or yellow produce, (3) white, tan or brown produce, and (4) green produce group? NOTE: for yes, the salad bar must have one produce of each of the color groups listed above.
◯ Yes     ◯ No
26. Are 3 or more healthy-fat salad dressings offered along with separate olive oil and vinegar as salad dressing options?
◯ Yes     ◯ No
Grab-n-Go, Kiosk, Truck, Portable Meals for Off-Site Consumption
27. How many Grab-n-Go sandwiches are available with whole grain bread products or wraps?
◯ 0     ◯ 1     ◯ 2     ◯ ≥3 ◯ NA—no Grab-n-Go
28. How many Grab-n-Go sandwiches are made with lean meat options, such as turkey, ham, chicken, tuna, or peanut butter?
◯ 0     ◯ 1     ◯ 2     ◯ ≥3 ◯ NA—no Grab-n-Go
29. How many pre-assembled salads are available with a variety of dark leafy greens and colorful fresh veggie options?
◯ 0     ◯ 1     ◯ 2     ◯ ≥3 ◯ NA—no Grab-n-Go
30. How many healthier snacks/side options are available at the Grab-n-Go?
◯ 0     ◯ 1     ◯ 2     ◯ ≥3 ◯ NA—no Grab-n-Go
BEHAVIORAL DESIGN
31. Are there current posters, information stands and hand-out centers, banners, electronic message boards, flyers and other health messaging materials prominently located at the main patron entrance?
◯ Yes     ◯ No
32. Is there current signage promoting healthy choices within the Dining Facility?
◯ Yes     ◯ No
33. Are healthy choices labeled (green-coded or healthy icon) on food displays within the Dining Facility?
◯ Yes—all or most labeled accurately   ◯ Yes—some labeled accurately
◯ No—none labeled
34. Are “Green Coded food items” placed on the hot line and elsewhere as the first/most prominent selection available in the category, such as hot vegetables, starchy sides, and proteins? ◯ Yes     ◯ No
35. Is one grilled, fresh vegetable or hot vegetable served at the short order station? NA—for no short order in operation
◯ Yes     ◯ No     ◯ N/A
36. Reviewing the options on the main line and short order line, are there any deep fat fried items being served (French Fries, main entrees, vegetables and starches)
◯ No deep fried items served (short order or main line)     ◯ Yes, only 1 item
◯ Yes, two or more items
37. Is there at least one healthy “Featured Meal” promoted with a sample plate, photograph, or sign at the point of choice?
◯ Yes     ◯ No
38. Where are desserts located within the dining facility?
◯ Accessible in the serving area    ◯ Out of view and past the main line
◯ Out of view and not easily accessible
39. If chilled display cabinets are available, is there at least one cut up fruit option available in dessert section at lunch and dinner? If no chilled display is available, are diners encouraged at the salad bar and at the dessert bar
◯ Yes     ◯ No

### Appendix A.2

Community Assessment Form
COMMUNITY
1. Does the installation or base have a cross-functional Nutritional Environment Working Group working to improve the food environment on the installation/base?
Membership may include: dietitian, health promotion coordinator, the Appropriated Fund (APF) dining facility, exchange, commissary, Non-Appropriated Fund [NAF] food facilities, lodging, fitness center, and any other installation stakeholders interested in promoting a healthy nutritional environment. AFI 48-103, Army CR2C, Navy Blue H organizations
◯ Yes     ◯ No
2. Is the Nutritional Environment Working Group conducting an assessment of the installation/base nutritional environment at least annually?
NA = No work group
◯ Yes     ◯ No     ◯ NA
3. Is the Nutritional Environment Working Group developing an annual healthy food improvement action plan?
NA = No work group
◯ Yes     ◯ No     ◯ NA
4. Is the Nutritional Environment Working Group briefing installation or base leadership at least annually?
NA = No work group
◯ Yes     ◯ No     ◯ NA
5. Does the Nutritional Environment Working Group explore viability of locally sourced food? Locally sourced food may include: promotions with Commissary, farmers markets, community gardens or community supported agriculture programs?
NA = No work group
◯ Yes     ◯ No     ◯ NA
6. Does the installation or base have a marketing/media plan to promote healthy food sources for the installation/base?
◯ Yes     ◯ No
7. Does the installation/base have a farmer’s market?
Points are bonus points and added to the score not taken away
◯ Yes     ◯ No
8. Does the installation/base have a community garden?
Points are bonus points and added to the score not taken away
◯ Yes     ◯ No
9. Does the installation/base have a working group to address areas considered to be food deserts and strategies to support those areas?
◯ Yes     ◯ No

### Appendix A.3

Worksite Assessment Form
WORKSITE
1. Does the Senior Mission Commander (Highest ranking officer on the installation or base) have a “healthy worksite” policy (or philosophy statement) promoting healthy food and beverage choices in the work environment? NA Applies if Garrison/Base Commander is the highest ranking officer
◯ Yes     ◯ No     ◯ NA
2. Does the Senior Mission Commander worksite have a written policy/statement which makes healthier food and beverage choices available during meetings, events, or any other work gatherings where food is served? (For example, does the policy include instructions for following nutrition guidelines for food and beverages offered at these events?) NA applies if Garrison/Base Commander is the highest ranking officer
◯ Yes     ◯ No     ◯ NA
3. Does the Garrison or Base Commander have a “healthy worksite” policy (or philosophy statement) promoting healthy food and beverage choices in the work environment?
◯ Yes     ◯ No   
4. Does the Garrison or Base Commander worksite have a written policy/statement which makes healthier food and beverage choices available during meetings, events, or any other work gatherings where food is served? (For example, does the policy include instructions for following nutrition guidelines for food and beverages offered at these events?)
◯ Yes     ◯ No    
5. Does the Medical Treatment Facility Commander have a “healthy worksite” policy (or philosophy statement) promoting healthy food and beverage choices in the work environment?
◯ Yes     ◯ No
6. Does the Medical Treatment Facility Commander worksite have a written policy/statement which makes healthier food and beverage choices available during meetings, events, or any other work gatherings where food is served? (For example, does the policy include instructions for following nutrition guidelines for food and beverages offered at these events?)
◯ Yes     ◯ No

### Appendix A.4

Fast-Food Assessment Form
FAST-FOOD BUILDING
1. Is the restaurant located in a free standing building?
◯ Yes    ◯ No—it is part of a food court
◯ No—it shares the building with another restaurant (part of a duplex)
◯ No—it shares the building with more than one restaurant/store (part of a strip mall)
◯ No—it shares the building or space in some other capacity
FOOD POLICY
2. Is the calorie information clearly visible to customers on the menu board?
◯ Yes     ◯ No
FOOD AVAILABILITY
3. How many healthier entree options (meat or vegetarian), including salads, are available?
Count each option separately, except low fat deli meats counts as 1 option.
Healthier main meal options include: skinless chicken/turkey breast, low-fat deli meat (chicken, turkey, ham), veggie/turkey burgers, fish/seafood, pork top loin, beef top round steak or roast, items that is not prepared by breading or frying, and is not covered in sauce or salad dressing
Healthier items are described as: baked, grilled, sautéed, stir fried, steamed
◯ 0     ◯ 1–2     ◯ 3–4     ◯ 5–7  ◯ ≥8
4. How many different healthier side options are available? Side is defined as a single serving of a food that may accompany a meal or entree or eaten on its own.
Healthier side options include: prepared side salad (not from a salad bar), fruit without added sugar/syrup, baked/steamed vegetables, legume (beans) without added fat, brown/wild rice, quinoa, couscous, barley, and or whole grain pasta.
Healthier items are described as: baked, grilled, sautéed, stir fried, steamed
◯ 0     ◯ 1–2     ◯ 3–4     ◯ 5–7  ◯ ≥8
5. How many whole grain bread/tortilla wrap options are available?
Whole grain options include: wheat, oats, barley, rye
◯ 0     ◯ 1     ◯ 2     ◯ ≥3
6. How many healthier grab-and-go snack options are available (near the cash register)?
Healthier Grab-n-Go option can include:
Granola/protein bars with low-sugar or no sugar added (<200 kcals)
Plain nuts/seeds and/or dried fruit/nut trail mixes without added sugar, candy, or chocolate (<200 kcals)
Dried fruit without added sugar (<200 kcals) and/or canned/packaged fruit in its own juice or water
Baked pre-packaged snacks (chips, pretzels) (<200 kcals)
Hummus, hard-boiled eggs, fresh veggies, whole fruit, etc.
◯ 0     ◯ 1–2     ◯ 3–4     ◯ ≥5     ◯NA
7. How many different healthy beverage options are available?
Healthy beverages are defined as:
Beverages with ≤25 calories per 8 oz service (exclude fruit juice and milk)
Water, including flavored, carbonated, and seltzer water
100% juice–fruit or vegetable juice with no added sugar
Fat free or 1% milk (both flavored or unflavored)
Fortified soy, almond, or rice milk with <12 g sugar per serving
◯ 0     ◯ 1–5     ◯ 6–10     ◯ 11–15   ◯≥16
BEHAVIORAL DESIGN
8. Inside the establishment, are there posters or signs on the wall that promote healthier options? NA—For COVID conditions
◯ Yes     ◯ No     ◯ NA
9. Are there healthier options (fresh fruit, baked chips, trail mix) available at the point of sale (customer side of the counter)? NA—For COVID conditions
◯ Yes     ◯ No     ◯ NA
10. Are healthier menu options identified (as “healthier” or with a logo) on menu boards or menus?
◯ Yes     ◯ No
11. Are healthier options listed first for their category on the menu board or menu?
Example: Healthier side items (such as fresh fruit) are listed first in the “side” section
◯ Yes     ◯ No    
12. With a combo/value meal, can you substitute the less healthy default (fries or chips) for a healthier option (side salad or fruit) at no cost?
◯ Yes     ◯ No     ◯ NA– no combo meals
13. Is health messaging/promotion of healthier substitutions emphasized (at no cost) and highly visible at the point of service?
◯ Yes     ◯ No
14. Is additional nutrition information (more than calories) available upon request by the customer?
Either on the website, in a handout, or booklet/binder
◯ Yes     ◯ No
15. Are the “healthier” options priced similar to the other foods?
◯ Yes     ◯ No
16. Are the “healthier” items advertised or featured on a rotational basis?
◯ Yes     ◯ No
